# Thymus antibody-secreting cells: once forgotten but not lost

**DOI:** 10.3389/fimmu.2023.1170438

**Published:** 2023-04-12

**Authors:** KimAnh Trang Pioli, Peter Dion Pioli

**Affiliations:** Department of Biochemistry, Microbiology and Immunology, College of Medicine, University of Saskatchewan, Saskatoon, SK, Canada

**Keywords:** antibody-secreting cell, plasmablast, plasma cell, thymus, aging, autoimmunity, interferon, toll-like receptor 7

## Abstract

Antibody-secreting cells are essential contributors to the humoral response. This is due to multiple factors which include: 1) the ability to secrete thousands of antibodies per second, 2) the ability to regulate the immune response and 3) the potential to be long-lived. Not surprisingly, these cells can be found in numerous sites within the body which include organs that directly interface with potential pathogens (e.g., gut) and others that provide long-term survival niches (e.g., bone marrow). Even though antibody-secreting cells were first identified in the thymus of both humans and rodents in the 1960s, if not earlier, only recently has this population begun to be extensively investigated. In this article, we provide an update regarding the current breath of knowledge pertaining to thymus antibody-secreting cells and discuss the potential roles of these cells and their impact on health.

## 1 Introduction

Following their activation, B cells can undergo terminal differentiation into antibody-secreting cells (ASCs) (1). The context in which this occurs can subsequently regulate the type of ASC being produced. For example, innate-like B cells which include both marginal zone and B1 B cells can be readily activated in a T cell independent manner which preferentially results in the production of IgM secreting ASCs with a short-lived proliferative, or plasmablast (PB), phenotype (1). In contrast, follicular B cells are generally engaged in T cell dependent responses which can include extrafollicular (EF) as well as germinal center (GC)-derived production of ASCs. The EF pathway kinetically proceeds the GC and generates PBs which display low levels of antibody (Ab) affinity maturation (1, 2). B cells which enter the GC pathway undergo extensive somatic hypermutation producing ASCs which secrete Abs with high affinity for their target antigen and possess a mature, post-mitotic plasma cell (PC) phenotype (1, 2). While the pathways described above are skewed towards a particular type of ASC response, all 3 can result in ASCs that secrete class switched Abs and ultimately differentiate into a mature, potentially long-lived, phenotype (3–7).

The functionality ascribed to ASCs has expanded extensively in recent years (8). For example, ASCs have been shown to suppress inflammation in multiple disease settings through their production of cytokines which include interleukin (IL)-10 and IL-35 (9–13) thus leading to their inclusion within the global regulatory B cell population (14, 15). However, the ability of these cells to impact the immune response can also be mediated through direct cellular interactions (16). Further extending the sphere of ASC influence, these cells have been shown to regulate aspects of bone marrow (BM) hematopoiesis in both humans (17) and mice (18, 19). As hematopoiesis is a continual and life-long process, these studies have shed light on the critical importance of ASCs in every-day homeostatic functions. With that being said, ASCs will always be considered an essential component of the humoral immune system due largely to their production of antigen-specific Abs as well as their potential to be long-lived (20).

Mechanistically, Abs provide protection through multiple avenues such as opsonization, antibody-dependent cellular cytotoxicity and neutralization which can nullify the ability of invading pathogens to infect and propagate within the host (21). This benefit can quickly become a detriment when ASCs produce Abs that recognize self-antigens in the form of nucleic acids [e.g., double-stranded DNA (22, 23)] or proteins [e.g., insulin (24)]. While much consideration has been given to circulating ASCs in the context of autoimmunity, it is known throughout the field that the THY serves as a reservoir for these cells in multiple autoimmune diseases (25–27). Additionally, the presence of ectopic GCs in the autoimmune THY further points to this organ as being a source of generation for autoreactive ASCs (27–29). On that note, thymectomy has been used as a method of treatment in diseases such as myasthenia gravis (MG) to eliminate production of newly formed autoreactive ASCs (30, 31). While thymectomy has some benefit, it is not entirely curative as autoreactive THY B cell clones can escape the THY and be maintained in the periphery of MG patients (32). In considering how THY ASCs might contribute to MG and autoimmunity in general, it is critical to understand how these cells are limited in their production, maintenance and function under healthy conditions. By disrupting these basic mechanisms and recapitulating various autoimmune-related pathologies, we have the potential to identify druggable targets/pathways. While the identification of THY ASCs first occurred at least as early as the 1960s (25, 27, 33), only recently has that there has been a push to study THY ASCs in both human and mouse species with the above goals in mind (34–37). This review summarizes the current state of the field regarding THY ASCs and discusses future directions in understanding the etiology and function of these cells.

## 2 Current knowledge in the field

### 2.1 Location

In the THY, ASCs are incredibly rare and consist of less than 0.05% of total organ cellularity (37). Not surprisingly, the attempt to localize these cells within particular THY niches has been difficult. Initial characterization *via* light microscopy demonstrated the presence of ASCs within the THY medulla with them mostly residing in local perivascular spaces (25). These observations have since been confirmed with the use of more advanced imaging techniques. Interestingly enough, there is some discordance between species as THY ASCs in humans are primarily confined to medullary perivascular spaces (34, 36) versus those in mice which can be found throughout the THY medulla and in close proximity to both B and T cells (35). It is unclear if these discrepancies are a result of limited sampling in humans or are representative of greater biological meaning such as differences in function or circulatory phenotypes.

The chemokine cues required to localize ASCs within the THY have not been experimentally determined. However, THY ASCs do express CXCR4 and CXCR3 (35, 37) and their respective ligands, CXCL12 and CXCL9/10/11, can be found in the THY (38). Important to note, CXCL12 is highly enriched in the THY cortex (39) whereas CXCL9/10/11 are found more so in the medulla (38) suggesting that these factors may compete with one another and dictate the local positioning of THY ASCs. Recent work which examined trafficking of CD8 T cells, demonstrated the loss of these cells in the THY of *Cxcl10*^-/-^ mice (40) supporting the importance of this chemokine in maintaining selected populations within the THY. Furthermore, increased expression of CD69 and CD44 has been observed on mouse ASCs from the THY when compared to those from the BM and SPL (35, 37) suggesting that these cells possess a tissue residency phenotype (41) and remain segregated within the THY. Recent intravital imaging experiments demonstrated that BM ASCs were not completely sessile as they periodically migrated within the BM compartment (42). This behavior was attributed to competing signals from VLA-4 (promoting adhesion) and CXCR4 (promoting migration) (42). Undoubtedly, it will be interesting to see how deletion of these receptors or other integrin and chemokine receptors in ASCs alters their positioning, or more globally, their retention within the THY.

### 2.2 Production

Substantial evidence supports the existence of THY B cell progenitors which mature into fully functional B cells *in situ* (43, 44). While the full Ab repertoire of these cells is unknown, autoreactive B cells have been identified in both the human and mouse THY even under healthy conditions (45, 46). Upon first glance, this seems inherently detrimental; however, multiple studies based in mice have revealed the ability of autoreactive THY B cells to regulate negative selection of CD4 single positive T cells (45, 47–49) and to augment the production of regulatory T cells (50, 51). Thus, the presence of autoreactive THY B cells seems to be a necessary component to systemic immune tolerance. During this tolerogenic process, THY B cells undergo AID dependent class switching (45, 47) indicative of a productive immune response. Data indicate this requires cognate B cell-T cell interactions as class switching was abrogated upon deletion of major histocompatibility complex (MHC) II and CD40 as well as in T cell receptor (TCR) transgenic mice whose CD4 T cells were specific for ovalbumin (45). This raises the possibility that activated THY B cells may also differentiate into potentially autoreactive ASCs.

In support of the above, parabiosis and intravenous antibody labeling experiments in mice have indicated local production of THY ASCs (35, 37). While these types of functional experiments are missing in humans, *in vitro* differentiation experiments using human samples have shown the ability of THY B cells to produce ASCs (34, 36). B cells can be activated and terminally differentiated into ASCs upon receiving either T cell independent or dependent stimuli (1). Recent studies from our lab as well as others have ascertained a role for T cell derived signals in the production of THY ASCs (35, 37). Using αCD154(CD40L) blocking Abs, we observed a reduction in proliferating THY B cells as well as PBs in C57BL/6 mice that were given αCD154 Abs for either 2- or 4-weeks (37). Complementary work using DO11.10 BALB/c and OT-II C57BL/6 TCR transgenic mice further corroborated the importance of T cell help for the generation of THY ASCs (35). It is known that T cell dependent ASC production can occur through both EF and GC routes (2, 52). While ectopic GCs have been observed in the autoimmune THY (27–29, 53), this does not appear to be a major route of THY ASC production under healthy conditions. This conclusion is supported by experiments that used *Icosl*^-/-^ and CD4-Cre *Bcl6*^fl/fl^ mice which lacked functional T follicular helper (T_FH_) cell signals yet still produced normal amounts of THY ASCs (35). This observation contrasted with ASCs in the Peyer’s patches and spleen (SPL) which were both reduced upon genetic ablation of T_FH_ signals. While T cell help clearly contributes to the production of THY ASCs, the residual presence of these cells in the absence of T cell signals suggests that a T cell independent pathway also feeds into the generation of THY ASCs (35, 37). When compared to B cells from the SPL, those isolated from the THY were minimally responsive to lipopolysaccharide (54). However, THY B cells can proliferate upon exposure to the Toll-like receptor (TLR) 7/8 agonist, R848, in the presence of IL-2 (36). Along these lines, B cells in the THY possess increased expression of TLR7 relative to those from the SPL and BM (37). Rather interestingly, the same is true for ASCs found in the THY (37).

The above paragraph discussed stimulatory cues and accessory signals required for THY ASC generation. However, it is currently not known which B cell populations (e.g., follicular, B-1) differentiate into THY ASCs. Earlier experiments that profiled fetal and neonatal mouse THY B cells observed a population dominated by expression of CD5 (55), a B-1a B cell-associated cell surface marker (56). In contrast, THY B cells from older animals were more mixed in that a substantial proportion was CD5^-^ (55) and presumably of the B-2 (i.e., follicular) B cell lineage. Adding further support for the presence of B-2 B cells, phenotyping of adult mice identified THY B cells which expressed IgD as well as CD23 (45, 48, 57, 58). Along these lines, both fetal liver- and BM-derived progenitors can produce mature CD19^+^ CD45R(B220)^+^ THY B cells when transplanted into Rag2^-/-^ hosts leading to the conclusion that both B-1 and B-2 B cells can mature in the THY (48) and potentially differentiate into ASCs. Unfortunately, assessment of ASC production was not included in these analyses. Ultimately, the best way to determine which THY B cell populations generate ASCs might rely on their purification *via* cell surface phenotypes and subsequent differentiation into ASCs. *In vitro* stimulation experiments might reveal ASC generative potential as shown for neonatal THY CD19^+^ CD70^+^ CD138^-^ B cells (34). However, physiologically relevant answers may only be provided by direct THY injection of purified B cell populations followed by tracking their ASC differentiation using congenic markers or fluorescent reporters. In this *in vivo* setting, THY B cell populations would be exposed to biological relevant signals. Overall, further experiments will be needed to better understand how different stimulatory signals and types of B cells give rise to various THY ASC populations, and how these different populations contribute to host physiology and disease.

### 2.3 VDJ landscape and immunoglobulin repertoire

As ASCs are built for Ab production (59, 60), a major question regarding those in the non-autoimmune THY pertains to the specificities of the Abs that they produce. Not surprisingly, VDJ sequencing has been a popular tool in attempting to address this question. These analyses (34, 35, 37) have pointed to some basic commonalities between THY ASCs from humans and mice. In general, both species possess an extensive amount of class switching to a wide variety of isotypes. In mice, this is influenced by strain as BALB/c THY ASCs are significantly enriched for IgE (35) whereas this isotype is essentially absent in ASCs isolated from the THY of C57BL/6 mice (35, 37). A reduced contribution of IgE has also been observed for ASCs in the human THY (34). In both species, class switched THY ASCs appear early in life (34–36) and, at least in mice, this is not driven by the presence of commensal microbiota (35). Upon examination of clonotypes, mouse THY ASCs were shown to be highly diverse (37) and were not dominated by hyperexpanded clones that would be selected for in response to a single immunizing agent. While sample size was limited, THY ASC clonotypes demonstrated minimal overlap with ASCs from BM and SPL which suggested limited circulation of these cells, at least to the organs assessed (37). In agreement with the lack of GC requirements, mouse THY ASCs possessed low levels of mutations in their complementarity determining regions 1 and 2 (35). Similar results were observed in humans; however, albeit with some level of donor-to-donor variability (34).

Currently, the specificities of THY ASC Abs are yet to be characterized in mice. However, studies in humans revealed that the THY ASC population possessed cells that were reactive to viruses (36) and bacteria (34). That being said, these assessments were far from comprehensive and most likely did not reflect all potential specificities to both foreign and self. Given the presence of autoreactive B cells in the THY, the observation that THY ASCs share similar patterns of IGHV gene usage with their upstream B cell progenitors (34) may indicate the presence, at least transiently, of auto-Ab producing ASCs. Accordingly, a subset of human THY ASCs and B cells were both shown to use the V_H_ 4-34 gene segment (34) which is associated with autoreactivity (61, 62). Further complicating the assessment of THY ASC reactivities is the phenomenon of polyreactivity in which Abs can recognize both foreign as well as self-antigens due to similarities between antigenic epitopes (62, 63). Therefore, the assumption that a particular ASC only recognizes a single antigenic target is a dangerous oversimplification.

### 2.4 Thymus antibody-secreting cell transcriptional signature: function and regulation

Recent single cell RNA-sequencing (scRNA-seq) experiments in humans (34) and mice (35, 37) have provided clues as to the function and regulation of these cells. In mice, the data suggest a potential role in antigen presentation as supported by upregulation of various MHC genes (35, 37) such as *H2-Aa*, *H2-Ab1*, *H2-D1*, *H2-K1*, *H2-Q7* and *H2-T22* as well as increased MHC II surface expression (35, 37). When compared to those from the BM, THY ASCs also possessed increased expression of *Cd74* and *Pdia3*. *Cd74* encodes for the invariant chain which acts as an MHC II chaperone (64). Additionally, this gene product plays a role in B cell survival and can be upregulated *via* B cell-T cell interactions (65) while *Pdia3* is essential for proper expression of MHC I (66). If THY ASCs were to act as antigen-presenting cells, then they would require access to self-antigens through intrinsic or extrinsic routes. Accordingly, mouse THY ASCs have been shown to express the Aire protein (35) in addition to membrane, or cell surface, immunoglobulin (35, 37) suggesting multiple mechanisms in which these cells can procure self-antigen for presentation to developing T cells. In this sense, Aire can drive intrinsic expression of tissue-restricted antigens which are then subsequently presented *via* peptide-MHC complexes (49, 57, 67). In contrast, membrane immunoglobulin would allow autoreactive ASCs to extrinsically acquire autoantigens presumably released to the environment by apoptotic or necrotic cells (63). These antigens would then be internalized, processed and subsequently presented to CD4 T cells *via* MHC II molecules.

In considering how THY ASCs may be regulated, gene ontology comparisons of differentially expressed genes between mouse THY ASCs and their BM and SPL counterparts have been informative. These analyses indicated that THY ASCs were functionally skewed towards innate and viral responses and that this was most likely driven by interferon (IFN) signaling. In support of this, THY ASCs were shown to express multiple IFN receptor genes (e.g., *Ifnar1*, *Ifnar2*) and a number of interferon responsive genes (68) (e.g., *Isg15, Stat1, Tlr7*) *via* scRNA-seq (37). Interestingly, some of the protein products of these genes including TLR7, Ly-6C, CD69 and MHC II were upregulated by ASCs as well as upstream CD19^+^ CD45R(B220)^+^ CD138^-^ B cells in the mouse THY (37). While the above data were from C57BL/6 mice, some of these targets (Ly-6C, MHC II) have also been confirmed in THY ASCs from the BALB/c strain (35). Furthermore, recent work has shown the ability of Type III IFN, and to a lesser extent Type II IFN, to regulate aspects of mouse THY B cell biology such as Ly-6C and CXCR3 expression (58). Upon closer examination of ASCs from the human THY, a subset of cells expressed CD69 and MHC II-related HLA-DQA1. Perhaps not surprisingly, human THY B cells also possessed an interferon responsive signature as indicated by increased expression of genes such as IFITM1, IFIT3 and OAS2 (34). Pertinent to this discussion, Type I, Type II and Type III IFNs have all been shown to play regulatory roles regarding B cell activation and ASC production (69–71).

Currently, little is known about the longevity of THY ASCs, although flow cytometric phenotyping in mice has shown this population to possess a high percentage of Ki-67 expressing CD19^+^ CD45R(B220)^+^ cells (37). This phenotype aligns well with a PB identity and most likely denotes a limited lifespan for this population (72). In support of this, inhibition of CD154 signals resulted in a precipitous loss of this population within 2 weeks of treatment. Furthermore, pseudotime analysis of scRNA-seq data yielded the identification of 3 putative ASC lineages (37). Two of these lineages were similar with heightened expression of canonical ASC genes such as *Jchain* and *Mzb1* which suggested a continued maturation. In contrast, the 3^rd^ lineage formed the majority of THY ASCs and did not demonstrate the same pattern of maturation. Rather, this lineage downregulated genes related to ribosomal function/protein production (e.g., *Rps9*, *Rpl3*) as well as survival (e.g., *Birc5*) perhaps foretelling a shortened lifespan.

**Figure 1:**
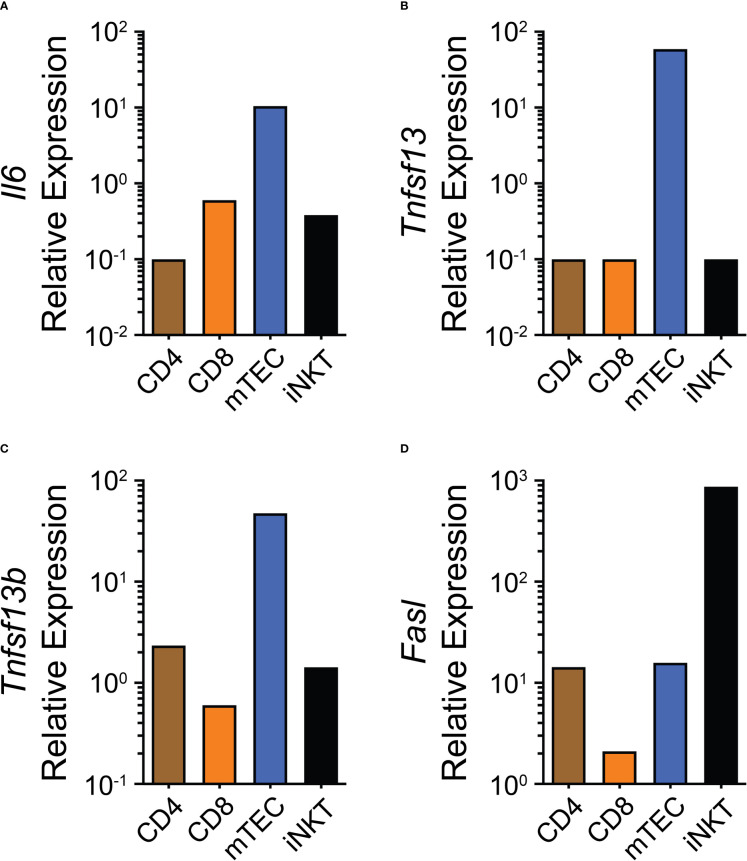
Expression of potential thymus antibody-secreting cell niche regulatory factors. **(A–D)** Gene expression data from the publicly available ImmGen database showing **(A)** *Il6*, **(B)** *Tnfsf13*, **(C)** *Tnfsf13b* and **(D)** *Fasl* for CD4, CD8, mTEC and iNKT cells.

If THY ASCs are indeed short-lived then this could be due to multiple causes. For example, they may not have access to adequate survival signals; however, this seems rather unlikely as human THY epithelial cells have been shown to secrete IL-6 (73). Furthermore, mouse medullary thymic epithelial cells (mTECs) transcriptionally express *Il6*, *Tnfsf13* and *Tnfsf13b* as found in the publicly available Immunological Genome Project (ImmGen) database (**Figures 1A–C**). The latter 2 genes encode for A Proliferation-Inducing Ligand (APRIL) and B Cell Activating Factor (BAFF), respectively, and all 3 cytokines contribute to the survival niche of ASCs (74). Alternatively, and not necessarily mutually exclusive, active cell death mechanisms may be in place which limit the production and/or survival of THY ASCs. One such possibility is the death receptor CD95(Fas) which can be upregulated on the surface of B cells following stimulation through CD40 (75, 76). Current data suggest that B cells actively receive CD154:CD40 signaling in the THY as genetic ablation of CD40 expression led to the loss of THY B cell class switching (45) and antibody-mediated CD154 blockade resulted in reduced proliferation of THY B cells (37). Along these lines, we have observed high expression of CD95(Fas) on the surface of “resting” THY B cells which was enhanced by *in vivo* treatment with CD40 agonistic Abs (37). While we have not directly tested the role of CD95(Fas) in limiting the expansion of THY B cell and ASCs, it is well known that CD178(FasL):CD95(Fas) interactions modulate THY T cell development (77) as well as the survival of autoreactive B cells (78). Furthermore, multiple populations in the THY such as mTECs, CD4 T cells and invariant natural killer T (iNKT) cells express *Fasl* which encodes for CD178(FasL) (ImmGen, **Figure 1D**). Importantly, IFNγ can increase CD95(Fas) expression by developing thymocytes (79) and also by peripheral B cells from individuals infected with human immunodeficiency virus (80). As such, IFNs may play a critical role in finetuning ASC production and function in mice and possibly also humans.

### 2.5 Aging

ASCs can be produced under homeostatic conditions and have the potential to be long-lived; therefore, it is no surprise that they have been found to accumulate over the course of aging in organs such as the SPL and BM (19, 42). Examination of the human THY has suggested a similar phenotype in which ASCs increased as a fraction of total THY (36). This was mainly due to the enhanced production of IgG and IgA class switched ASCs. Recent work in mice revealed that this observation may be dictated by sex as ASCs accumulated in the THY of middle-aged (12 months old) Prdm1-eYFP male mice relative to their younger (3 months old) counterparts (37). In contrast, this did not occur in females. The reasons behind this disparity are not yet known; however, ASC numbers in the young female THY were already equivalent to those observed in middle-aged males (37). This indicates that the THY may have an upper limit in ASC carrying capacity that females, at least in mice, reach at a younger age.

As discussed above, the function of THY ASCs is poorly understood possibly resulting from the chronological period in which these cells have been analyzed. For example, PCs from the BM augmented myelopoiesis from hematopoietic stem cells in an age-dependent manner (19). In that study, old BM PCs adopted a pro-inflammatory phenotype and contributed to the ability of BM stromal cells to express inflammatory factors such as *Il1b* (19). Accordingly, THY dendritic cells have shown age-associated increases in the expression of factors such as *Il1a*, *Il1b*, *Il6* and *Tnf* (81) making it tempting to speculate that old THY ASCs may contribute to this process. Supporting a potential age-associated shift in THY ASC function, analysis of selected proteins *via* flow cytometry displayed alterations in THY ASC expression of CD69 with this surface receptor being reduced with age (37). Furthermore, THY B cells have demonstrated age-associated changes such as reduced Aire expression (57). As such, any phenotypic changes in ASCs may be a result of *de novo* alterations or alternatively, inheritance from their upstream B cell progenitors. Regarding the effects of aging, one must always consider the overall contributions of intrinsic cellular age as well as the extrinsic aged environment (82). Therefore, it will be important not only to understand age-associated differences in THY ASCs but also how these changes are influenced by the above factors. Would adoptive transfer of THY ASCs from young (e.g., 3 months) mice into old (e.g., 17-19 months) recipients result in “old” THY ASCs as represented by changes in gene expression or a functional readout such as per cell cytokine secretion? Alternatively, using Cre-mediated fluorescence reporter timestamping (83), do 2-months old and 4-months old ASCs behave differently even if isolated concurrently from the same animal?

## 3 Looking towards the future: insights into autoimmunity?

**Figure 2:**
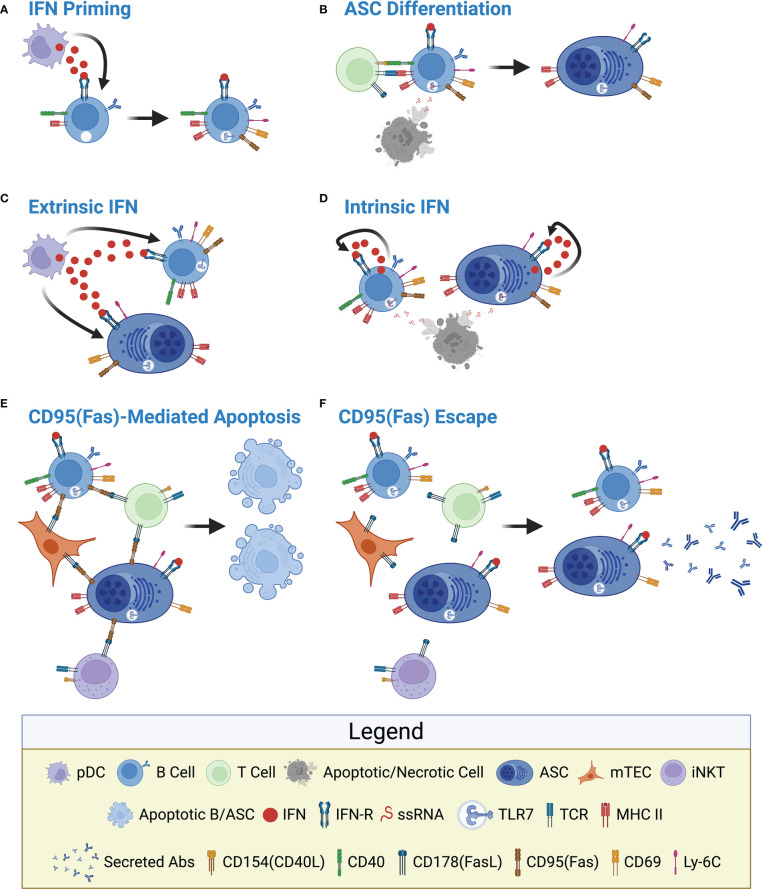
Hypothetical model depicting interferon-mediated regulation of thymus B cells and antibody-secreting cells. **(A)** IFN Priming: IFNs secreted by cells such as pDCs directly induce expression of TLR7, CD69, CD95(Fas), Ly-6C and MHC II by THY B cells. This could be driven by Type I, II and/or III IFNs collectively referred to as IFN. The various IFN receptors are commonly referred to as IFN-R for simplicity. **(B)** ASC Differentiation: In conjunction with CD40 signaling, TLR7 activation stimulates THY B cell proliferation and differentiation into ASCs independent of antigen specificity. In this model, ASCs initially inherent IFN-induced proteins from their upstream B cell precursors. ssRNA, the ligand for TLR7, is provided by locally apoptotic/necrotic cells. **(C)** Extrinsic IFN: The IFN responsive phenotype is maintained by extrinsic IFNs provided by cells such as pDCs. **(D)** Intrinsic IFN: As a result of TLR7 stimulation, THY B cells and ASCs produce their own IFNs which signal autonomously to reinforce the IFN responsive phenotype. **(E)** CD95(Fas)-Mediated Apoptosis: As a negative feedback mechanism, CD178(FasL) expressed by multiple cells within the THY medulla induces CD95(Fas)-mediated apoptosis of potentially autoreactive THY B cells and ASCs. Potential sources of CD178(FasL) include mTECs, iNKT cells as well as CD4 T cells. **(F)** CD95(Fas) Escape: Loss of CD95(Fas) signaling through receptor downregulation or by other means results in the survival of THY B cells and ASCs. This provides a long-term depot for auto-Ab producing THY ASCs. Figure created with BioRender.

From the human and mouse studies described above, we can begin to develop a hypothetical regulatory pathway which would limit the production and survival of autoreactive ASCs within the THY (**Figure 2**). Previous work has demonstrated the constitutive expression of both Type I and Type II IFNs within the THY (84–87) and these pathways have been shown to be functionally relevant in the THY medulla (86, 88) where THY B cells and ASCs are known to reside. In addition, Type III IFN was recently shown to modulate the ability of THY B cells to induce regulatory T cell generation (58). As such, we propose that IFNs, possibly secreted by THY plasmacytoid dendritic cells (pDCs) (84, 89, 90), directly act upon THY B cells driving the upregulation of TLR7, CD69, CD95(Fas), Ly-6C and MHC II (**Figure 2A**), all of which are IFN responsive (69, 91–93). These B cells then directly interact with developing CD4 T cells *via* CD154:CD40 inducing Aire in THY B cells and the ability to express tissue-restricted antigens (57, 67) that are then processed and presented to CD4 T cells *via* MHC II. These events ultimately lead to the negative selection, or apoptosis, of cognate autoreactive T cells (45, 47, 48). In this scenario, THY B cells do not expand nor produce significant amounts of THY ASCs as supported by the inability of *in vivo* αCD40 treatment to increase these THY populations (37). However, upon exposure to ligands such as single stranded RNA (ssRNA), TLR7 could provide an additional stimulatory signal which would cooperate with CD40 stimulation resulting in THY B cell proliferation and differentiation into ASCs (**Figure 2B**). This idea is consistent with past studies demonstrating the ability of TLR signals to augment B cell responses (94–96). As thymocyte apoptosis regulates the production of regulatory T cells (97), it is plausible that ssRNA is released to the extracellular environment during this process. In addition, reactivation of endogenous retroviruses within the THY could also provide the requisite TLR ligands (98, 99). Subsequently, the IFN responsive phenotype can be maintained by extrinsically derived IFNs (e.g., pDCs) (**Figure 2C**) or alternatively, by IFNs intrinsically produced by TLR7 stimulated THY B cells and ASCs (**Figure 2D**). This latter pathway would be reminiscent to what was observed when SPL B cells from wildtype and IFNAR1^-/-^ mice were stimulated *in vitro* with the TLR7 ligand, R848 (100). In those experiments, an IFN-dependent positive feedback loop maintained high TLR7 expression and responsiveness (100). In the above scenarios, IFN stimulation provides a built-in kill switch through the induction of CD95(Fas) expression on the surface of THY B cells and, presumably, THY ASCs. Subsequent interactions with CD178(FasL) expressing cells within the THY medulla would lead to the elimination of B cells and ASCs in the THY thus preventing their accumulation (78) (**Figure 2E**). This effect would be aided by their expression of CD69 which would act to prolong their exposure to apoptotic signals through the suppression of S1P_1_ mediated THY egress (93).

So how is the above model hypothetical relevant to autoimmunity? Several autoimmune diseases possess alterations in the THY that include B cell activation, GC formation and/or increased production of ASCs (27–29, 43, 53). Importantly, this leads to functional consequences including destruction of the THY microenvironment as well as production of auto-Abs which promote cellular pathology and loss of organ function (26, 29, 43, 53). This is perhaps not surprising given that the THY is inherently enriched for B cells with autoreactivity (46, 48).

The observation that THY B cells express high levels of TLR7 is critical as it provides a plausible mechanism that would allow for the activation and differentiation of THY B cells in the absence of direct B cell receptor stimulation. In other words, autoreactive B cells would be responsive in the absence of their cognate self-antigen allowing for the production of ASCs that secrete a wide repertoire of auto-Abs. The importance of TLR7 in autoimmunity is well appreciated (101–105) and this receptor is hypothesized to play a role in the MG THY following infection with Epstein-Barr virus (EBV) (106, 107). However, the role of TLR7 in the autoreactive THY could be more widespread given the already high levels of TLR7 expression by THY B cells in the absence of overt infection (37).

While the potential importance of TLR7 cannot be understated, the key to understanding the B cell autoimmune response within the THY may lie in factors that regulate apoptosis as well as localization. For instance, CD95(Fas) signaling would theoretically “mop up” autoreactive B cells and ASCs that were generated within the THY (**Figure 2E**). However, if this failsafe was lost due to gene mutation (108–110) or other forms of regulation (111), then pathogenic THY B cells and ASCs would be allowed to survive (**Figure 2F**) and potentially escape the THY. Interestingly, alterations in CD95(Fas) signaling have been observed in aging (112, 113). Furthermore, we have demonstrated decreased THY ASC expression of CD69 with age (37) which may facilitate their thymic egress and subsequent localization into more optimal survival niches. This could partially explain why thymectomy is not always a successful treatment in individuals with MG (30, 32). While this regulatory loop remains to be tested, it provides an example of how defining THY ASC phenotypes and nodes of regulation in health may lead to key breakthroughs in understanding the etiology of autoimmune diseases that possess a significant THY B cell/ASC component.

## 4 Summary

We have presented the current state of the field regarding what is known about THY ASCs in terms of both their production and what their function(s) might be independent of Ab secretion. While these cells may play a role in allergic tolerance *via* homeostatic IgE production (35), we have largely focused on how the data derived from studies in healthy mice and humans may provide insights into potential nodes of regulation that are disrupted in autoimmunity. We have observed high TLR7 expression in THY B cells and ASCs compared to those from the BM and SPL in both young and middle-aged mice (37). In this context, TLR7 in the THY compartment was equivalently expressed between both sexes. As such, the regulatory model we propose may explain why some autoimmune diseases show a loss of sex bias with aging (102). The THY has been proposed to play a key role in age-associated autoimmunity; however, this has mainly been attributed to disrupted T cell selection and production of regulatory T cells (114, 115). However, future experiments will be required to better understand how the loss of THY B cell and ASC regulation ultimately fits into the complex etiology of autoimmunity both from a general perspective as well as in a disease-specific manner.

## Data availability statement

The original contributions presented in the study are included in the article/supplementary material. Further inquiries can be directed to the corresponding author.

## Author contributions

KP and PP conceptualized, wrote and edited the manuscript. All authors contributed to the article and approved the submitted version.

## References

[1] Nutt SL Hodgkin PD Tarlinton DM Corcoran LM. The generation of antibody-secreting plasma cells. Nat Rev Immunol (2015) 15(3):160–71. doi: 10.1038/nri3795 25698678

[2] Elsner RA Shlomchik MJ. Germinal center and extrafollicular b cell responses in vaccination, immunity, and autoimmunity. Immunity (2020) 53(6):1136–50. doi: 10.1016/j.immuni.2020.11.006 PMC7748291 33326765

[3] Bergqvist P Stensson A Lycke NY Bemark M. T Cell-independent IgA class switch recombination is restricted to the GALT and occurs prior to manifest germinal center formation. J Immunol (2010) 184(7):3545–53. doi: 10.4049/jimmunol.0901895 20207993

[4] Bortnick A Chernova I Quinn WJ 3rd Mugnier M Cancro MP Allman D. Long-lived bone marrow plasma cells are induced early in response to T cell-independent or T cell-dependent antigens. J Immunol (2012) 188(11):5389–96. doi: 10.4049/jimmunol.1102808 PMC4341991 22529295

[5] Good-Jacobson KL Szumilas CG Chen L Sharpe AH Tomayko MM Shlomchik MJ. PD-1 regulates germinal center b cell survival and the formation and affinity of long-lived plasma cells. Nat Immunol (2010) 11(6):535–42. doi: 10.1038/ni.1877 PMC2874069 20453843

[6] Hsu MC Toellner KM Vinuesa CG Maclennan IC. B cell clones that sustain long-term plasmablast growth in T-independent extrafollicular antibody responses. Proc Natl Acad Sci USA (2006) 103(15):5905–10. doi: 10.1073/pnas.0601502103 PMC1424660 16585532

[7] Ottens K Schneider J Kane LP Satterthwaite AB. PIK3IP1 promotes extrafollicular class switching in T-dependent immune responses. J Immunol (2020) 205(8):2100–8. doi: 10.4049/jimmunol.2000584 PMC7541772 32887751

[8] Pioli PD. Plasma cells, the next generation: Beyond antibody secretion. Front Immunol (2019) 10:2768. doi: 10.3389/fimmu.2019.02768 31824518 PMC6883717

[9] Lino AC Dang VD Lampropoulou V Welle A Joedicke J Pohar J. LAG-3 inhibitory receptor expression identifies immunosuppressive natural regulatory plasma cells. Immunity. (2018) 49(1):120–33.e9. doi: 10.1016/j.immuni.2018.06.007 30005826 PMC6057275

[10] Matsumoto M Baba A Yokota T Nishikawa H Ohkawa Y Kayama H. Interleukin-10-producing plasmablasts exert regulatory function in autoimmune inflammation. Immunity. (2014) 41(6):1040–51. doi: 10.1016/j.immuni.2014.10.016 25484301

[11] Rojas OL Probstel AK Porfilio EA Wang AA Charabati M Sun T. Recirculating intestinal IgA-producing cells regulate neuroinflammation *via* IL-10. Cell. (2019) 177(2):492–3. doi: 10.1016/j.cell.2019.03.037 30951673

[12] Shalapour S Font-Burgada J Di Caro G Zhong Z Sanchez-Lopez E Dhar D. Immunosuppressive plasma cells impede T-cell-dependent immunogenic chemotherapy. Nature. (2015) 521(7550):94–8. doi: 10.1038/nature14395 PMC4501632 25924065

[13] Shen P Roch T Lampropoulou V O'Connor RA Stervbo U Hilgenberg E. IL-35-producing b cells are critical regulators of immunity during autoimmune and infectious diseases. Nature. (2014) 507(7492):366–70. doi: 10.1038/nature12979 PMC4260166 24572363

[14] Fillatreau S. Natural regulatory plasma cells. Curr Opin Immunol (2018) 55:62–6. doi: 10.1016/j.coi.2018.09.012 PMC6290076 30292126

[15] Fillatreau S. Regulatory functions of b cells and regulatory plasma cells. BioMed J (2019) 42(4):233–42. doi: 10.1016/j.bj.2019.05.008 PMC6818159 31627865

[16] Pelletier N McHeyzer-Williams LJ Wong KA Urich E Fazilleau N McHeyzer-Williams MG. Plasma cells negatively regulate the follicular helper T cell program. Nat Immunol (2010) 11(12):1110–8. doi: 10.1038/ni.1954 PMC3058870 21037578

[17] Tsujimoto T Lisukov IA Huang N Mahmoud MS Kawano MM. Plasma cells induce apoptosis of pre-b cells by interacting with bone marrow stromal cells. Blood. (1996) 87(8):3375–83. doi: 10.1182/blood.V87.8.3375.bloodjournal8783375 8605355

[18] Meng L Almeida LN Clauder AK Lindemann T Luther J Link C. Bone marrow plasma cells modulate local myeloid-lineage differentiation *via* IL-10. Front Immunol (2019) 10:1183. doi: 10.3389/fimmu.2019.01183 31214168 PMC6555095

[19] Pioli PD Casero D Montecino-Rodriguez E Morrison SL Dorshkind K. Plasma cells are obligate effectors of enhanced myelopoiesis in aging bone marrow. Immunity. (2019) 51(2):351–66.e6. doi: 10.1016/j.immuni.2019.06.006 31303400 PMC6703913

[20] McGettigan SE Debes GF. Immunoregulation by antibody secreting cells in inflammation, infection, and cancer. Immunol Rev (2021) 303(1):103–18. doi: 10.1111/imr.12991 PMC8387433 34145601

[21] Forthal DN. Functions of antibodies. Microbiol Spectr (2014) 2(4):AID–0019-2014. doi: 10.1128/microbiolspec.AID-0019-2014 26104200

[22] Wang X Xia Y. Anti-double stranded DNA antibodies: Origin, pathogenicity, and targeted therapies. Front Immunol (2019) 10:1667. doi: 10.3389/fimmu.2019.01667 31379858 PMC6650533

[23] Wellmann U Letz M Herrmann M Angermuller S Kalden JR Winkler TH. The evolution of human anti-double-stranded DNA autoantibodies. Proc Natl Acad Sci United States America (2005) 102(26):9258–63. doi: 10.1073/pnas.0500132102 PMC1166593 15968001

[24] Burbelo PD Iadarola MJ Keller JM Warner BM. Autoantibodies targeting intracellular and extracellular proteins in autoimmunity. Front Immunol (2021) 12:548469. doi: 10.3389/fimmu.2021.548469 33763057 PMC7982651

[25] Goldstein G. Plasma cells in the human thymus. Aust J Exp Biol Med Sci (1966) 44(6):695–9. doi: 10.1038/icb.1966.66 4166152

[26] Hill ME Shiono H Newsom-Davis J Willcox N. The myasthenia gravis thymus: a rare source of human autoantibody-secreting plasma cells for testing potential therapeutics. J Neuroimmunol (2008) 201-202:50–6. doi: 10.1016/j.jneuroim.2008.06.027 18722675

[27] Mackay IR Degail P. Thymic "Germinal centres" and plasma cells in systemic lupus erythematosus. Lancet. (1963) 2(7309):667. doi: 10.1016/S0140-6736(63)90458-6 14052029

[28] Hidalgo Y Nunez S Fuenzalida MJ Flores-Santibanez F Saez PJ Dorner J. Thymic b cells promote germinal center-like structures and the expansion of follicular helper T cells in lupus-prone mice. Front Immunol (2020) 11:696. doi: 10.3389/fimmu.2020.00696 32411134 PMC7199236

[29] Sims GP Shiono H Willcox N Stott DI. Somatic hypermutation and selection of b cells in thymic germinal centers responding to acetylcholine receptor in myasthenia gravis. J Immunol (2001) 167(4):1935–44. doi: 10.4049/jimmunol.167.4.1935 11489973

[30] Lisak RP Richman DP. Thymectomy and myasthenia gravis. Proc Natl Acad Sci USA (2020) 117(51):32195–6. doi: 10.1073/pnas.2022901117 PMC7768708 33273117

[31] Wolfe GI Kaminski HJ Sonnett JR Aban IB Kuo HC Cutter GR. Randomized trial of thymectomy in myasthenia gravis. J Thorac Dis (2016) 8(12):E1782–E3. doi: 10.21037/jtd.2016.12.80 PMC5227194 28149641

[32] Jiang R Hoehn KB Lee CS Pham MC Homer RJ Detterbeck FC. Thymus-derived b cell clones persist in the circulation after thymectomy in myasthenia gravis. Proc Natl Acad Sci USA (2020) 117(48):30649–60. doi: 10.1073/pnas.2007206117 PMC7720237 33199596

[33] Sainte-Marie G. Plasmocytes in the thymus of the normal rat. J Immunol (1965) 94:172–6. 14253516

[34] Cordero H King RG Dogra P Dufeu C See SB Chong AM. Intrathymic differentiation of natural antibody-producing plasma cells in human neonates. Nat Commun (2021) 12(1):5761. doi: 10.1038/s41467-021-26069-2 34599177 PMC8486820

[35] Kwon DI Park ES Kim M Choi YH Lee MS Joo SH. Homeostatic serum IgE is secreted by plasma cells in the thymus and enhances mast cell survival. Nat Commun (2022) 13(1):1418. doi: 10.1038/s41467-022-29032-x 35301301 PMC8930980

[36] Nunez S Moore C Gao B Rogers K Hidalgo Y Del Nido PJ. The human thymus perivascular space is a functional niche for viral-specific plasma cells. Sci Immunol (2016) 1(6). doi: 10.1126/sciimmunol.aah4447 PMC5407379 28459117

[37] Pioli KT Lau KH Pioli PD. Thymus antibody-secreting cells possess an interferon gene signature and are preferentially expanded in young female mice. iScience. (2023) 26(3):106223. doi: 10.1016/j.isci.2023.106223 36890795 PMC9986522

[38] Bunting MD Comerford I McColl SR. Finding their niche: chemokines directing cell migration in the thymus. Immunol Cell Biol (2011) 89(2):185–96. doi: 10.1038/icb.2010.142 21135866

[39] Lucas B White AJ Parnell SM Henley PM Jenkinson WE Anderson G. Progressive changes in CXCR4 expression that define thymocyte positive selection are dispensable for both innate and conventional alphabetaT-cell development. Sci Rep (2017) 7(1):5068. doi: 10.1038/s41598-017-05182-7 28698642 PMC5505955

[40] Alanio C Barreira da Silva R Michonneau D Bousso P Ingersoll MA Albert ML. CXCR3/CXCL10 axis shapes tissue distribution of memory phenotype CD8(+) T cells in nonimmunized mice. J Immunol (2018) 200(1):139–46. doi: 10.4049/jimmunol.1700564 29187588

[41] Topham DJ Reilly EC. Tissue-resident memory CD8(+) T cells: From phenotype to function. Front Immunol (2018) 9:515. doi: 10.3389/fimmu.2018.00515 29632527 PMC5879098

[42] Benet Z Jing Z Fooksman DR. Plasma cell dynamics in the bone marrow niche. Cell Rep (2021) 34(6):108733. doi: 10.1016/j.celrep.2021.108733 33567286 PMC8023250

[43] Castaneda J Hidalgo Y Sauma D Rosemblatt M Bono MR Nunez S. The multifaceted roles of b cells in the thymus: From immune tolerance to autoimmunity. Front Immunol (2021) 12:766698. doi: 10.3389/fimmu.2021.766698 34790201 PMC8591215

[44] Perera J Huang H. The development and function of thymic b cells. Cell Mol Life Sci (2015) 72(14):2657–63. doi: 10.1007/s00018-015-1895-1 PMC4480206 25837998

[45] Perera J Zheng Z Li S Gudjonson H Kalinina O Benichou JIC. Self-Antigen-Driven thymic b cell class switching promotes T cell central tolerance. Cell Rep (2016) 17(2):387–98. doi: 10.1016/j.celrep.2016.09.011 PMC5091085 27705788

[46] Rother MB Schreurs MW Kroek R Bartol SJ van Dongen JJ van Zelm MC. The human thymus is enriched for autoreactive b cells. J Immunol (2016) 197(2):441–8. doi: 10.4049/jimmunol.1501992 27259853

[47] Lombard-Vadnais F Chabot-Roy G Zahn A Rodriguez Torres S Di Noia JM Melichar HJ. Activation-induced cytidine deaminase expression by thymic b cells promotes T-cell tolerance and limits autoimmunity. iScience (2023) 26(1):105852. doi: 10.1016/j.isci.2022.105852 36654860 PMC9840937

[48] Perera J Meng L Meng F Huang H. Autoreactive thymic b cells are efficient antigen-presenting cells of cognate self-antigens for T cell negative selection. Proc Natl Acad Sci United States America (2013) 110(42):17011–6. doi: 10.1073/pnas.1313001110 PMC3801014 24082098

[49] Yamano T Nedjic J Hinterberger M Steinert M Koser S Pinto S. Thymic b cells are licensed to present self antigens for central T cell tolerance induction. Immunity. (2015) 42(6):1048–61. doi: 10.1016/j.immuni.2015.05.013 26070482

[50] Lu FT Yang W Wang YH Ma HD Tang W Yang JB. Thymic b cells promote thymus-derived regulatory T cell development and proliferation. J Autoimmun (2015) 61:62–72. doi: 10.1016/j.jaut.2015.05.008 26071985

[51] Walters SN Webster KE Daley S Grey ST. A role for intrathymic b cells in the generation of natural regulatory T cells. J Immunol (2014) 193(1):170–6. doi: 10.4049/jimmunol.1302519 24872190

[52] Paus D Phan TG Chan TD Gardam S Basten A Brink R. Antigen recognition strength regulates the choice between extrafollicular plasma cell and germinal center b cell differentiation. J Exp Med (2006) 203(4):1081–91. doi: 10.1084/jem.20060087 PMC2118299 16606676

[53] Sarkkinen J Dunkel J Tuulasvaara A Huuskonen A Atula S Kekalainen E. Ectopic germinal centers in the thymus accurately predict prognosis of myasthenia gravis after thymectomy. Mod Pathol (2022) 35(9):1168–74. doi: 10.1038/s41379-022-01070-2 PMC9424113 35338262

[54] Inaba M Inaba K Adachi Y Nango K Ogata H Muramatsu S. Functional analyses of thymic CD5+ b cells. responsiveness to major histocompatibility complex class II-restricted T blasts but not to lipopolysaccharide or anti-IgM plus interleukin 4. J Exp Med (1990) 171(1):321–6. doi: 10.1084/jem.171.1.321 PMC2187665 1688610

[55] Nango K Inaba M Inaba K Adachi Y Than S Ishida T. Ontogeny of thymic b cells in normal mice. Cell Immunol (1991) 133(1):109–15. doi: 10.1016/0008-8749(91)90183-C 1703924

[56] Yoshimoto M. The ontogeny of murine b-1a cells. Int J hematology (2020) 111(5):622–7. doi: 10.1007/s12185-019-02787-8 PMC7247277 31802412

[57] Cepeda S Cantu C Orozco S Xiao Y Brown Z Semwal MK. Age-associated decline in thymic b cell expression of aire and aire-dependent self-antigens. Cell Rep (2018) 22(5):1276–87. doi: 10.1016/j.celrep.2018.01.015 PMC5813500 29386114

[58] Martinez RJ Breed ER Worota Y Ashby KM Voboril M Mathes T. Type III interferon drives thymic b cell activation and regulatory T cell generation. Proc Natl Acad Sci United States America (2023) 120(9):e2220120120. doi: 10.1073/pnas.2220120120 PMC9992806 36802427

[59] Gaudette BT Jones DD Bortnick A Argon Y Allman D. mTORC1 coordinates an immediate unfolded protein response-related transcriptome in activated b cells preceding antibody secretion. Nat Commun (2020) 11(1):723. doi: 10.1038/s41467-019-14032-1 32024827 PMC7002553

[60] Tellier J Shi W Minnich M Liao Y Crawford S Smyth GK. Blimp-1 controls plasma cell function through the regulation of immunoglobulin secretion and the unfolded protein response. Nat Immunol (2016) 17(3):323–30. doi: 10.1038/ni.3348 PMC4757736 26779600

[61] Pugh-Bernard AE Silverman GJ Cappione AJ Villano ME Ryan DH Insel RA. Regulation of inherently autoreactive VH4-34 b cells in the maintenance of human b cell tolerance. J Clin Invest (2001) 108(7):1061–70. doi: 10.1172/JCI200112462 PMC200949 11581307

[62] Schickel JN Glauzy S Ng YS Chamberlain N Massad C Isnardi I. Self-reactive VH4-34-expressing IgG b cells recognize commensal bacteria. J Exp Med (2017) 214(7):1991–2003. doi: 10.1084/jem.20160201 28500047 PMC5502416

[63] Suurmond J Diamond B. Autoantibodies in systemic autoimmune diseases: specificity and pathogenicity. J Clin Invest (2015) 125(6):2194–202. doi: 10.1172/JCI78084 PMC4497746 25938780

[64] Karakikes I Morrison IE O'Toole P Metodieva G Navarrete CV Gomez J. Interaction of HLA-DR and CD74 at the cell surface of antigen-presenting cells by single particle image analysis. FASEB J (2012) 26(12):4886–96. doi: 10.1096/fj.12-211466 22889831

[65] Radomir L Cohen S Kramer MP Bakos E Lewinsky H Barak A. T Cells regulate peripheral naive mature b cell survival by cell-cell contact mediated through SLAMF6 and SAP. J Immunol (2017) 199(8):2745–57. doi: 10.4049/jimmunol.1700557 PMC5805483 28904129

[66] Garbi N Tanaka S Momburg F Hammerling GJ. Impaired assembly of the major histocompatibility complex class I peptide-loading complex in mice deficient in the oxidoreductase ERp57. Nat Immunol (2006) 7(1):93–102. doi: 10.1038/ni1288 16311600

[67] Gies V Guffroy A Danion F Billaud P Keime C Fauny JD. B cells differentiate in human thymus and express AIRE. J Allergy Clin Immunol (2017) 139(3):1049–52.e12. doi: 10.1016/j.jaci.2016.09.044 27864026

[68] Rusinova I Forster S Yu S Kannan A Masse M Cumming H. Interferome v2.0: an updated database of annotated interferon-regulated genes. Nucleic Acids Res (2013) 41:D1040–6. doi: 10.1093/nar/gks1215 PMC3531205 23203888

[69] Jackson SW Jacobs HM Arkatkar T Dam EM Scharping NE Kolhatkar NS. B cell IFN-gamma receptor signaling promotes autoimmune germinal centers *via* cell-intrinsic induction of BCL-6. J Exp Med (2016) 213(5):733–50. doi: 10.1084/jem.20151724 PMC4854732 27069113

[70] Swanson CL Wilson TJ Strauch P Colonna M Pelanda R Torres RM. Type I IFN enhances follicular b cell contribution to the T cell-independent antibody response. J Exp Med (2010) 207(7):1485–500. doi: 10.1084/jem.20092695 PMC2901065 20566717

[71] Syedbasha M Bonfiglio F Linnik J Stuehler C Wuthrich D Egli A. Interferon-lambda enhances the differentiation of naive b cells into plasmablasts *via* the mTORC1 pathway. Cell Rep (2020) 33(1):108211. doi: 10.1016/j.celrep.2020.108211 33027651

[72] Pracht K Meinzinger J Daum P Schulz SR Reimer D Hauke M. A new staining protocol for detection of murine antibody-secreting plasma cell subsets by flow cytometry. Eur J Immunol (2017) 47(8):1389–92. doi: 10.1002/eji.201747019 28608550

[73] Le PT Lazorick S Whichard LP Yang YC Clark SC Haynes BF. Human thymic epithelial cells produce IL-6, granulocyte-monocyte-CSF, and leukemia inhibitory factor. J Immunol (1990) 145(10):3310–5. doi: 10.4049/jimmunol.145.10.3310 1700006

[74] Khodadadi L Cheng Q Radbruch A Hiepe F. The maintenance of memory plasma cells. Front Immunol (2019) 10:721. doi: 10.3389/fimmu.2019.00721 31024553 PMC6464033

[75] Schattner EJ Elkon KB Yoo DH Tumang J Krammer PH Crow MK. CD40 ligation induces apo-1/Fas expression on human b lymphocytes and facilitates apoptosis through the apo-1/Fas pathway. J Exp Med (1995) 182(5):1557–65. doi: 10.1084/jem.182.5.1557 PMC2192191 7595225

[76] Zhang X Li L Choe J Krajewski S Reed JC Thompson C. Up-regulation of bcl-xL expression protects CD40-activated human b cells from fas-mediated apoptosis. Cell Immunol (1996) 173(1):149–54. doi: 10.1006/cimm.1996.0260 8871610

[77] Castro JE Listman JA Jacobson BA Wang Y Lopez PA Ju S. Fas modulation of apoptosis during negative selection of thymocytes. Immunity (1996) 5(6):617–27. doi: 10.1016/S1074-7613(00)80275-7 8986720

[78] Rathmell JC Cooke MP Ho WY Grein J Townsend SE Davis MM. CD95 (Fas)-dependent elimination of self-reactive b cells upon interaction with CD4+ T cells. Nature. (1995) 376(6536):181–4. doi: 10.1038/376181a0 7603571

[79] Moulian N Bidault J Planche C Berrih-Aknin S. Two signaling pathways can increase fas expression in human thymocytes. Blood. (1998) 92(4):1297–307. doi: 10.1182/blood.V92.4.1297 9694718

[80] Sammicheli S Dang VP Ruffin N Pham HT Lantto R Vivar N. IL-7 promotes CD95-induced apoptosis in b cells *via* the IFN-gamma/STAT1 pathway. PloS One (2011) 6(12):e28629. doi: 10.1371/journal.pone.0028629 22194871 PMC3237470

[81] Ki S Park D Selden HJ Seita J Chung H Kim J. Global transcriptional profiling reveals distinct functions of thymic stromal subsets and age-related changes during thymic involution. Cell Rep (2014) 9(1):402–15. doi: 10.1016/j.celrep.2014.08.070 PMC4194175 25284794

[82] Dorshkind K Hofer T Montecino-Rodriguez E Pioli PD Rodewald HR. Do haematopoietic stem cells age? Nat Rev Immunol (2020) 20(3):196–202. doi: 10.1038/s41577-019-0236-2 31740804 PMC7879798

[83] Xu AQ Barbosa RR Calado DP. Genetic timestamping of plasma cells in vivo reveals tissue-specific homeostatic population turnover. eLife (2020) 9. doi: 10.7554/eLife.59850 PMC7682985 33136000

[84] Colantonio AD Epeldegui M Jesiak M Jachimowski L Blom B Uittenbogaart CH. IFN-alpha is constitutively expressed in the human thymus, but not in peripheral lymphoid organs. PloS One (2011) 6(8):e24252. doi: 10.1371/journal.pone.0024252 21904619 PMC3164161

[85] Lienenklaus S Cornitescu M Zietara N Lyszkiewicz M Gekara N Jablonska J. Novel reporter mouse reveals constitutive and inflammatory expression of IFN-beta *in vivo* . J Immunol (2009) 183(5):3229–36. doi: 10.4049/jimmunol.0804277 19667093

[86] Otero DC Baker DP David M. IRF7-dependent IFN-beta production in response to RANKL promotes medullary thymic epithelial cell development. J Immunol (2013) 190(7):3289–98. doi: 10.4049/jimmunol.1203086 PMC3608802 23440417

[87] Reynolds CJ Chong DLW Li Y Black SL Cutler A Webster Z. Bioluminescent reporting of *In vivo* IFN-gamma immune responses during infection and autoimmunity. J Immunol (2019) 202(8):2502–10. doi: 10.4049/jimmunol.1801453 PMC6452029 30814307

[88] Xing Y Wang X Jameson SC Hogquist KA. Late stages of T cell maturation in the thymus involve NF-kappaB and tonic type I interferon signaling. Nat Immunol (2016) 17(5):565–73. doi: 10.1038/ni.3419 PMC4837029 27043411

[89] Hadeiba H Lahl K Edalati A Oderup C Habtezion A Pachynski R. Plasmacytoid dendritic cells transport peripheral antigens to the thymus to promote central tolerance. Immunity. (2012) 36(3):438–50. doi: 10.1016/j.immuni.2012.01.017 PMC3315699 22444632

[90] Martin-Gayo E Sierra-Filardi E Corbi AL Toribio ML. Plasmacytoid dendritic cells resident in human thymus drive natural treg cell development. Blood (2010) 115(26):5366–75. doi: 10.1182/blood-2009-10-248260 20357241

[91] Kiefer K Oropallo MA Cancro MP Marshak-Rothstein A. Role of type I interferons in the activation of autoreactive b cells. Immunol Cell Biol (2012) 90(5):498–504. doi: 10.1038/icb.2012.10 22430248 PMC3701256

[92] Schlueter AJ Krieg AM de Vries P Li X. Type I interferon is the primary regulator of inducible ly-6C expression on T cells. J Interferon Cytokine Res Off J Int Soc Interferon Cytokine Res (2001) 21(8):621–9. doi: 10.1089/10799900152547885 11559440

[93] Shiow LR Rosen DB Brdickova N Xu Y An J Lanier LL. CD69 acts downstream of interferon-alpha/beta to inhibit S1P1 and lymphocyte egress from lymphoid organs. Nature. (2006) 440(7083):540–4. doi: 10.1038/nature04606 16525420

[94] Boeglin E Smulski CR Brun S Milosevic S Schneider P Fournel S. Toll-like receptor agonists synergize with CD40L to induce either proliferation or plasma cell differentiation of mouse b cells. PloS One (2011) 6(10):e25542. doi: 10.1371/journal.pone.0025542 21991317 PMC3184999

[95] Pone EJ Zhang J Mai T White CA Li G Sakakura JK. BCR-signalling synergizes with TLR-signalling for induction of AID and immunoglobulin class-switching through the non-canonical NF-kappaB pathway. Nat Commun (2012) 3:767. doi: 10.1038/ncomms1769 22473011 PMC3337981

[96] Suthers AN Sarantopoulos S. TLR7/TLR9- and b cell receptor-signaling crosstalk: Promotion of potentially dangerous b cells. Front Immunol (2017) 8:775. doi: 10.3389/fimmu.2017.00775 28751890 PMC5507964

[97] Konkel JE Jin W Abbatiello B Grainger JR Chen W. Thymocyte apoptosis drives the intrathymic generation of regulatory T cells. Proc Natl Acad Sci USA (2014) 111(4):E465–73. doi: 10.1073/pnas.1320319111 PMC3910656 24474796

[98] Passos V Pires AR Foxall RB Nunes-Cabaço H Sousa AE. Expression of human endogenous retroviruses in the human thymus along T cell development. Front Virol (2022) 2. doi: 10.3389/fviro.2022.826393

[99] Yu P Lubben W Slomka H Gebler J Konert M Cai C. Nucleic acid-sensing toll-like receptors are essential for the control of endogenous retrovirus viremia and ERV-induced tumors. Immunity (2012) 37(5):867–79. doi: 10.1016/j.immuni.2012.07.018 23142781

[100] Green NM Laws A Kiefer K Busconi L Kim YM Brinkmann MM. Murine b cell response to TLR7 ligands depends on an IFN-beta feedback loop. J Immunol (2009) 183(3):1569–76. doi: 10.4049/jimmunol.0803899 PMC2929820 19587008

[101] Brown GJ Canete PF Wang H Medhavy A Bones J Roco JA. TLR7 gain-of-function genetic variation causes human lupus. Nature (2022) 605(7909):349–56. doi: 10.1038/s41586-022-04642-z PMC9095492 35477763

[102] Dodd KC Menon M. Sex bias in lymphocytes: Implications for autoimmune diseases. Front Immunol (2022) 13:945762. doi: 10.3389/fimmu.2022.945762 36505451 PMC9730535

[103] Fillatreau S Manfroi B Dorner T. Toll-like receptor signalling in b cells during systemic lupus erythematosus. Nat Rev Rheumatol (2021) 17(2):98–108. doi: 10.1038/s41584-020-00544-4 33339987 PMC7747191

[104] Souyris M Cenac C Azar P Daviaud D Canivet A Grunenwald S. TLR7 escapes X chromosome inactivation in immune cells. Sci Immunol (2018) 3(19). doi: 10.1126/sciimmunol.aap8855 29374079

[105] Souyris M Mejia JE Chaumeil J Guery JC. Female predisposition to TLR7-driven autoimmunity: gene dosage and the escape from X chromosome inactivation. Semin Immunopathol (2019) 41(2):153–64. doi: 10.1007/s00281-018-0712-y 30276444

[106] Cavalcante P Barzago C Baggi F Antozzi C Maggi L Mantegazza R. Toll-like receptors 7 and 9 in myasthenia gravis thymus: amplifiers of autoimmunity? Ann New York Acad Sci (2018) 1413(1):11–24. doi: 10.1111/nyas.13534 29363775

[107] Cavalcante P Galbardi B Franzi S Marcuzzo S Barzago C Bonanno S. Increased expression of toll-like receptors 7 and 9 in myasthenia gravis thymus characterized by active Epstein-Barr virus infection. Immunobiology (2016) 221(4):516–27. doi: 10.1016/j.imbio.2015.12.007 26723518

[108] Al-Sakran LH Marrie RA Blackburn DF Knox KB Evans CD. Establishing the incidence and prevalence of multiple sclerosis in Saskatchewan. Can J Neurol Sci (2018) 45(3):295–303. doi: 10.1017/cjn.2017.301 29557321

[109] Rieux-Laucat F Le Deist F Fischer A. Autoimmune lymphoproliferative syndromes: genetic defects of apoptosis pathways. Cell Death Differ (2003) 10(1):124–33. doi: 10.1038/sj.cdd.4401190 12655301

[110] Seyrek K Ivanisenko NV Wohlfromm F Espe J Lavrik IN. Impact of human CD95 mutations on cell death and autoimmunity: a model. Trends Immunol (2022) 43(1):22–40. doi: 10.1016/j.it.2021.11.006 34872845

[111] Koncz G Hueber AO. The Fas/CD95 receptor regulates the death of autoreactive b cells and the selection of antigen-specific b cells. Front Immunol (2012) 3:207. doi: 10.3389/fimmu.2012.00207 22848207 PMC3404404

[112] Wallach-Dayan SB Petukhov D Ahdut-HaCohen R Richter-Dayan M Breuer R. sFasL-the key to a riddle: Immune responses in aging lung and disease. Int J Mol Sci (2021) 22(4):2177. doi: 10.3390/ijms22042177 33671651 PMC7926921

[113] Zhao H Roychoudhury J Doggett TA Apte RS Ferguson TA. Age-dependent changes in FasL (CD95L) modulate macrophage function in a model of age-related macular degeneration. Invest Ophthalmol Visual Sci (2013) 54(8):5321–31. doi: 10.1167/iovs.13-12122 PMC3738220 23821188

[114] Palmer DB. The effect of age on thymic function. Front Immunol (2013) 4:316. doi: 10.3389/fimmu.2013.00316 24109481 PMC3791471

[115] Rose NR. Thymus function, ageing and autoimmunity. Immunol Lett (1994) 40(3):225–30. doi: 10.1016/0165-2478(94)00060-3 7959891

